# PRO—biotics? Are pre- and probiotics a valuable adjunct to fluoridated toothpaste in the battle against dental decay?

**DOI:** 10.1038/s41432-024-00976-x

**Published:** 2024-01-31

**Authors:** Rhyanna Mughal

**Affiliations:** Specialty Dentist in Paediatric Dentistry, Leeds Dental Institute, Leeds, UK

**Keywords:** Dental caries, Diseases

## Abstract

**Design:**

An investigator and participant masked, parallel-group randomised control trial examining the tooth surfaces of 288 children aged 5–9 years (*n* = 141 in the intervention group, *n* = 147 in the control group). Children in the intervention group were provided a daily lozenge containing 2% arginine (prebiotic), *Lacticaseibacillus rhamnosus* and *Lactobacillus paracasei* subsp. *paracasei* (probiotics).

Children in the control group were provided with a placebo lozenge. Parents of participants were also provided with 1450 ppm fluoride toothpaste and advised to brush their children’s teeth twice daily. Clinical and radiographic examinations were undertaken at baseline and 10–12 months assessing caries activity, progression and regression at a tooth surface level. A modified ICDAS and radiographic scoring system were used to record presence and extent of carious lesions.

**Case selection:**

Of 343 children who met the inclusion criteria, 21,888 tooth surfaces were examined in 288 low caries risk children aged between 5–9 years. Fifty-four participants withdrew consent (*n* = 31 for intervention group, *n* = 24 for control group) and 1 child was excluded as they were pre-cooperative for their dental examination. Participants were recruited from four municipalities in Denmark. To be eligible to participate, children had to be medically healthy, cooperative for clinical and radiographic examination, and able to ingest a daily lozenge.

**Data analysis:**

The primary outcome for this study was relative risk reduction (RRR) of caries activity, progression and regression. Although not present in this study, the main study broadly showed equality between the two groups in terms of their social demographic, dietary, oral health-related factors. The authors used modified Poisson regression to determine any surface level differences between the intervention and placebo groups. The threshold for statistical significance was set as follows: *p* < 0.05 was considered statistically significant. The authors describe that a power calculation was undertaken for the main study. However, none of the outcomes in this study were powered for.

**Results:**

19,950 tooth surfaces were included in the final analysis. There was no significant difference in change in caries activity between the two groups. There was a trend towards reduction in relative risk in the intervention group, with fewer active lesions (RRR: 15.3%; −6.0%, −32.4%), more caries regression (RRR: 0.3%; −0.4%, −1.0%) and reduced caries progression (RRR: 13.6%; −8.0%, −30.9%) observed than in the control group.

**Conclusions:**

The use of a probiotic and prebiotic daily lozenges as an adjunct to 1450 ppm fluoride toothpaste and oral hygiene instruction did not result in a significantly reduced relative risk of change in caries status compared to placebo. Further research over an increased intervention time with a higher caries risk population may identify potential advantages of adjunctive pre- and probiotics fluoride toothpaste in the prevention, arrest and regression of dental caries.

## A Commentary on

**Pørksen C J, Ekstrand K R, Markvart M, Larsen T, Garrido L E, Bakhshandeh A**.

The efficacy of combined arginine and probiotics as an add-on to 1450 ppm fluoride toothpaste to prevent and control dental caries in children – A randomized controlled trial. *J Dent* 2023; **137:** 104670.

**GRADE Rating:** 

## Commentary

The global burden of dental caries is extensive affecting more than one third of the world’s population^1^. Dental caries has an estimated prevalence of 43%, making it a major public health concern^1^. Caries is a multifactorial disease, influenced by factors including diet, oral hygiene and the oral environment. A range of public health guidelines have been developed, aimed at governments, dental health professionals and the general population. In England, the Department of Health and Social Care issued “Delivering better oral health: an evidence-based toolkit for prevention publication (2021)”^2^. These interventions focus on dietary changes, good oral hygiene practice and professional preventive interventions, such as regular topical fluoride application. Since its introduction in the late 1970s, the use of fluoridated toothpaste has been credited as the primary component in contributing to reduction of caries incidence^3^. Despite extensive investment in nationwide programmes such as “SMILE4LIFE” and “Starting Well Core: 0–2 s”, and the shift in focus to preventative dentistry, dental caries remains the most prevalent non-communicable disease worldwide^1,4^. In recent years, research has explored other topical or systemic products that may act as an adjunct to fluoride toothpaste by counteracting the demineralising activity of the caries microbiome and/or encouraging remineralisation, helping to reduce or slow caries progression and development^3^.

This randomised controlled trial (RCT) looks at the use of a combination of probiotics (*Lacticaseibacillus rhamnosus* and *Lactobacillus paracasei* subsp. *paracasei*) and a prebiotic as an adjunct to 1450 ppm fluoridate toothpaste. Oral bacteria metabolise arginine, resulting in the production of ammonia, thus raising the pH of the biofilm^5^. The probiotics act directly on the oral biofilm using a variety of mechanisms, such as bacteriocin production and growth inhibition to neutralise the effect of cariogenic bacteria^6^. This is a secondary analysis of data from a previously published trial by the same authors. This earlier trial demonstrated a statistically significant effect on caries increment with a lozenge combining prebiotic arginine and probiotics. This secondary analysis sought to focus on the effect of the same intervention on progression, arrest and regression of pre-existing caries lesions.

The aim of the study was to observe the efficacy of ingested arginine and probiotics on caries progression, regression and lesion activity at a tooth surface level over a period of 10–12 months. Participants’ parents completed a survey at baseline and follow up reviews to enable consideration of potentially confounding factors such as dietary habits; frequency of brushing, and parental education level. Both the investigators and participants were masked to allocation by the use of placebo pro/prebiotic capsules, to ensure that potential bias introduced by participant behavioural changes or caries detection by investigators was mitigated.

Because this is a secondary analysis of trial data, there is no power calculations for the outcomes reported. Therefore, findings should be interpreted with caution. The inclusion criteria are documented in the “main study”, requiring children to be medically fit and well, between the ages of 5 and 9 years and reside in one of the four described municipalities of Denmark^5^. Additionally, all participants were low caries risk. This may reduce the generalisability of the results and potential intervention to demonstrate a clinically important effect, although may have been a pragmatic decision to ensure a compliant, homogeneous participant cohort.

Although the investigators were trained and calibrated in the use of the original ICDAS scoring system, they were not calibrated for the use of the modified classifications used in this analysis^7,8^. Furthermore, the examiners excluded surfaces affected by molar-incisor hypoplasia (MIH) which could lead to an underestimation of caries progression as these are typically high-risk surfaces.

Compliance of ingestion of the lozenges was measured throughout the study, however they relied upon weekly and monthly electronic Patient Reported Outcomes. This is subject to reporting bias, as parents may have reported falsely elevated levels of compliance. The investigators considered adequate compliance to be consumption of the lozenges at least 5 days out of every 7 (~70%). Overall compliance was 87.5%, which is high. This highlights the importance of patient compliance. Of note, it might be expected that compliance rates may be lower in higher caries risk populations which may impact the effectiveness of the intervention.

Is there a gap in the market for pro- and prebiotics as an adjunct to fluoride? Ultimately, no significant difference was found between the intervention and placebo group in this study. Therefore, the surface level reduction in caries progression rate, increased regression rate and lower number of active carious lesions described by the authors should be interpreted with caution. While adjunctive prebiotic and probiotic lozenges show promise in reducing caries increment in low caries risk children, there is no apparent effect on pre-existing carious lesions. An adequately powered study in a high caries risk group may reveal over a longer time period may reveal further utility of adjunctive pro- and prebiotics in the management of caries.

Practice points

- There is benefit of adjunctive pre- and probiotic daily lozenges alongside fluoride toothpaste and twice daily brushing in the management of pre-existing carious lesions.
- Regular examinations, twice daily brushing with fluoride toothpaste, reduced frequency of ingestion of cariogenic food and drink, and regular topical fluoride application continue to be the most effective methods for caries prevention, arrest and regression.

## References

[1] Global oral health status report: towards universal health coverage for oral health by 2030. Geneva: World Health Organization; 2022. Licence: CC BY-NC-SA 3.0 IGO.

[2] Public Health England, Department of Health and Social Care, NHSE&I. (2021): Delivering Better Oral Health – an evidence-based toolkit for prevention. https://www.gov.uk/government/publications/delivering-better-oral-health-anevidence-based-toolkit-for-prevention.

[3] Pitts NB, Zero DT, Marsh PD, Ekstrand K, Weintraub JA, Ramos-Gomez F (2017). Dental caries. Nat Rev Dis Primers.

[4] Rodriguez A, Biazus-Dalcin C, Marshall J, Gorman R. Smile4life: a co-designed educational and training resource guide. University of Dundee: NHS Education for Scotland. 2022.

[5] Pørksen CJ, Keller MK, Damholt A, Frederiksen AK, Ekstrand KR, Markvart M (2023). The effect of a lozenge combining prebiotic arginine and probiotics on caries increment in children during 10–12 months, a randomized clinical trial. J Dent.

[6] Twetman S, Jørgensen MR, Keller MK (2017). Fifteen years of probiotic therapy in the dental context: what has been achieved?. J Calif Dent Assoc.

[7] Ekstrand KR, Gimenez T, Ferreira FR, Mendes FM, Braga MM (2018). The international caries detection and assessment system–ICDAS: a systematic review. Caries Res.

[8] Ekstrand KR, Luna LE, Promisiero L, Cortes A, Cuevas S, Reyes JF (2011). The reliability and accuracy of two methods for proximal caries detection and depth on directly visible proximal surfaces: an in vitro study. Caries Res.

